# Effects of a stepwise alveolar recruitment maneuver on lung volume distribution in dogs assessed by computed tomography

**DOI:** 10.3389/fvets.2023.1232635

**Published:** 2024-01-16

**Authors:** Ana Flávia Sanchez, Aline Magalhães Ambrósio, Ana Carolina B. C. Fonseca Pinto, Marco Aurélio Amador Pereira, Felipe Silveira Rego Monteiro Andrade, Renata Ramos Rodrigues, Alessandro Rodrigues de Carvalho Martins, Carina Outi Baroni, Bruno Ferrante, Denise Tabacchi Fantoni

**Affiliations:** ^1^Department of Surgery, School of Veterinary Medicine and Animal Science, University of São Paulo, São Paulo, São Paulo, Brazil; ^2^Department of Veterinary Clinical Sciences, Diagnostic Imaging Purdue University College of Veterinary Medicine, West Lafayette, IN, United States

**Keywords:** alveolar recruitment maneuver, atelectasis, mechanical ventilation, computed tomography, anesthesia, imaging techniques, lung compliance, alveoli

## Abstract

**Background:**

Pulmonary atelectasis is a commonly occurs during anesthesia. In these cases, mechanical ventilation (MV) associated with alveolar recruitment maneuvers (ARMs) and positive end-expiratory pressure (PEEP) is indicated to reverse the condition, ensure adequate gas exchange and improve oxygenation. ARMs can trigger volutrauma, barotrauma, and atelectrauma. Therefore, computed tomography (CT) is the gold-standard method for monitoring lung aeration after ARM.

**Objective:**

To evaluate lung volume distribution after stepwise ARMs using computed tomography (CT).

**Methods:**

Twelve dogs weighing 24.0 ± 6.0 kg, aged 3 ± 1 years, of both sexes and different breeds, underwent orchiectomy or ovariohysterectomy. The animals were anesthetized and ventilated in volume-controlled mode. ARMs were then initiated by positive end-expiratory pressure (PEEP) titration (5, 10, 15, and 20 cmH_2_O). CT scans, cardiovascular parameters, and ventilatory mechanics were evaluated at all time points. Data were assessed for normality using the Shapiro–Wilk test and a two-way analysis of variance, followed by a *post-hoc* Bonferroni test to identify differences between time points. Statistical significance was attributed to a value of *p* of <0.05.

**Results:**

CT demonstrated that the ARMs increased ventilation throughout the lung, including the dependent regions, with volumes that increased and decreased proportionally with PEEP titration. When they reached PEEP 10 and 5 cmH_2_O descending (d), they remained significantly higher than those in PEEP 0 cmH_2_O (baseline). Static compliance improved about 40% at PEEP 10d and PEEP 5d compared to baseline. There was an increase in heart rate (HR) from PEEP 15 increasing (i) (74.5%) to PEEP 10d (54.8%) compared to baseline. Mean arterial blood pressure (MABP) decreased approximately 9% from PEEP 15i to PEEP 15d compared to baseline.

**Conclusion:**

Lung attenuation and regional and global volumes assessed by CT showed that maximum pulmonary aeration distribution followed by PEEP titration occurred at PEEP 20 cmH_2_O, maintaining the lungs normoaerated and without hyperaeration.

## 1 Introduction

Alveolar recruitment maneuvers (ARMs) have been extensively used. They provide homogeneous volume distribution in the pulmonary parenchyma and improve oxygenation and lung compliance by reversing atelectasis during anesthesia (1–3). The maneuvers frequently used promote high airway distension pressures for short periods, effectively improving oxygenation and lung mechanics; however, they can cause some degree of overdistention and, consequently, volutrauma, barotrauma, or atelectrauma.

CT is the gold standard for assessing the effects of ARMs on lung aeration in humans. Visual inspection is the most straightforward assessment method for evaluating increases in lung volume or attenuation. CT images show real-time dynamic lung performance at end-inspiration and expiration, allowing quantification of attenuation lung volume changes during ARM. Decreased volume and increased attenuation are related to atelectasis (anesthesia and decubitus), defined in CT images as a Hounsfield unit (HU) between −100 and +100 (4). Alveolar recruitment on CT is described as an increase in the aeration of previously non-aerated lung tissue, which is easily detectable and measured by lung recruitability (5). It is possible to evaluate the pulmonary distribution through the increase in gas volume using CT, as well as to verify the hyperdistended areas because the ARM performed by positive end-expiratory pressure (PEEP) can transform non-aerated lung areas (−100 to +100 HU) into poorly aerated regions (−101 to −500 HU), poorly aerated ones into normally aerated ones (−501 to −900 HU), and normally aerated ones into hyperaerated ones (−901 to −1,000 HU) (5).

In cats, CT has been used to evaluate the anesthetic protocols that promoted the best image quality and hemodynamic stability, quantify the effect of time and recumbency on CT measurements of lung volume and attenuation during general anesthesia, and evaluate pulmonary attenuation to detect overinflation at different pressure during controlled mechanical ventilation (4, 6–8). However, in anesthetized and mechanically ventilated dogs, the evaluation of lung attenuation by CT scans has been used to analyze the effects of two inspired oxygen concentrations on pulmonary aeration, to study the prevalence of lung atelectasis, and to assess the lung attenuation after ARMs to reverse lung atelectasis induced by anesthesia (9–11). Due to the lack of studies on this subject, the current study aimed to evaluate lung aeration distribution by helical CT and respiratory mechanics in dogs undergoing ARMs by PEEP titration and to identify optimal PEEP for aeration and the PEEP most likely to promote atelectasis and overdistention associated with respiratory mechanics. The study hypothesized that PEEP 20 cmH_2_O results in overinflation of nondependent lungs, and PEEP equal to or lower than 5 cmH_2_O during a stepwise decrease in PEEP results in atelectasis.

## 2 Materials and methods

### 2.1 Animals

This study was conducted at a Veterinary Teaching Hospital linked to the Faculty of Veterinary Medicine and Animal Science. The Institutional Ethics Committee on the Use of Animals approved the experimental protocol (n.5547200219). Twelve client-owned dogs weighing over 15 kg and aged between one and five years who underwent ovariohysterectomy or orchiectomy were included in this study. Obese dogs with a body condition score (BSC) > 6/9 and lean dogs with a BCS < 4/9 were excluded according to a validated scale (12). Only clinically healthy animals without a history of respiratory or cardiovascular disease and with no abnormalities on CT scans of the thorax, arterial blood gases, blood cell count, and renal and liver plasma chemistry panels were included in the study. The animals underwent ovariohysterectomy or orchiectomy at the end of the experimental protocol, and written informed consent was obtained from the client before entering any dog into the study protocol.

### 2.2 Anesthesia and monitoring

Food and water were withheld for 8 h and 4 h, respectively, before anesthesia. Acepromazine (0.03 mg kg^−1^; Syntec do Brasil, São Paulo, Brazil) associated with meperidine (3 mg kg^−1^; Dolosal; Cristália Produtos Químicos Farmacêuticos Ltda, São Paulo, Brazil) intramuscularly was administered as pre-anesthetic medication. After 15 min, a 20-gauge catheter (Angiocath; Becton Dickinson Indústrias Cirúrgicas, São Paulo, Brazil) was placed in the right cephalic vein, and 5 mg kg^−1^ propofol (Propovan; Cristália Produtos Químicos Farmacêuticos Ltda, São Paulo, Brazil) was injected intravenously (IV) for anesthesia induction. After orotracheal intubation, the endotracheal tube was connected to a microprocessor-controlled intensive care unit (ICU) ventilator, incorporating a calibrated variable orifice flow sensor pneumotachograph (G5; Hamilton Medical, Bonaduz, Switzerland). Anesthesia was maintained with continuous rate infusion (CRI) of propofol and remifentanil (0.2–0.4 mg kg^−1^ min^−1^ and 0.2–0.4 μg kg^−1^∙min^−1^, respectively) administered by an infusion pump (Agilia SP MC, Fresenius Kabi, Brazil). A bolus of rocuronium (0.6 mg kg^−1^, Esmeron; Organon do Brasil Indústria e Comércio Ltda, São Paulo, Brazil) was given following CRI administration (1 mg kg^−1^ h^−1^) to maintain mechanical ventilation. To ensure complete muscle paralysis, a nerve stimulator was placed over the ulnar nerves, and acceleromyography was used to assess the degree of neuromuscular blockade (TOF-Guard Biometer; Organon Teknika, São Paulo, Brazil). To avoid hypothermia, the animals were placed in dorsal recumbency on a heated mat with a blanket covering their bodies. To guarantee atelectasis at the beginning of the study, mechanical ventilation was maintained in the volume-controlled mode (7 mL. kg^−1^), zero end-expiratory pressure (PEEP 0 cmH_2_O), and FiO_2_:1.0 up to the first 30 min of anesthesia. An inspiration-expiration ratio (I: E) of 1:2 and the respiratory rate were adjusted to maintain PE’CO_2_ between 35 and 50 mmHg (5–7 kPa). A non-dispersive side stream infrared gas analyzer (POET IQ2-8500; Criticare System Inc., North Kingstown, RI, United States) was used for airway gases, which was calibrated before each experiment, and PE’CO_2_ was measured.

### 2.3 Experimental protocol

The ARMs consisted of PEEP titration with increments of 5 cmH_2_O beginning with 0 cmH_2_O, up to 20 cmH_2_O, followed by a step decreases of 5 cmH_2_O and ending with a PEEP 5 cmH_2_O. The FiO_2_ was changed from 1.0 to 0.4 at the beginning of the recruitment maneuver and was maintained until the end of the anesthetic procedure to avoid new atelectasis formation. The interval between each PEEP level was 5 min, and the entire maneuver lasted 35 min.

After completion of the protocol, the animals were sterilized in a operating room. Ten minutes before anesthesia ended, tramadol hydrochloride (3.0 mg kg^−1^ IV; Tramadol, Cristália Produtos Químicos Farmacêuticos Ltda, São Paulo, Brazil), dipyrone (25 mg kg^−1^ IV; Dipirona, IBASA, Porto Alegre, Brazil), and meloxicam (0.2 mg Kg^−1^ IV; Maxicam, Ourofino saúde Animal, Vinhedo, Brazil) were administered. Rocuronium infusion was stopped after the experiment and was reversed with neostigmine (0.04 mg kg^−1^, IV; Normastig, União Química, São Paulo, Brazil) associated with atropine (0.04 mg Kg^−1^, IV; Atrofarma, Farmace Indústria Químico Farmacêutica Cearense LTDA, Ceará, Brazil) after the end of surgery to avoid a residual block.

### 2.4 Data collection

#### 2.4.1 Computed tomography measurements

All CT scans were acquired using a 16-slice multidetector CT scanner (Philips Mx8000 IDT, Philips Healthcare, Best, Netherlands). CT images were obtained using helical acquisition, 120 kVp, 100 mAs, 16 × 15 mm collimation, 2 mm slice thickness, and a 512 × 5 12 matrix. The images were captured in a lung parenchyma window, window with a width of 1400 and center of 500 HU. The cuts were performed at the end of each PEEP step, and acquired during inspiratory and expiratory pauses lasting 10 s each. Pauses were performed manually using a mechanical ventilation device (G5 – Hamilton Medical, Bonaduz, Switzerland). After positioning the animal on the CT equipment table, a scout was performed, and the seventh intercostal space was selected.

##### 2.4.1.1 CT lung segmentation

**Figure 1:**
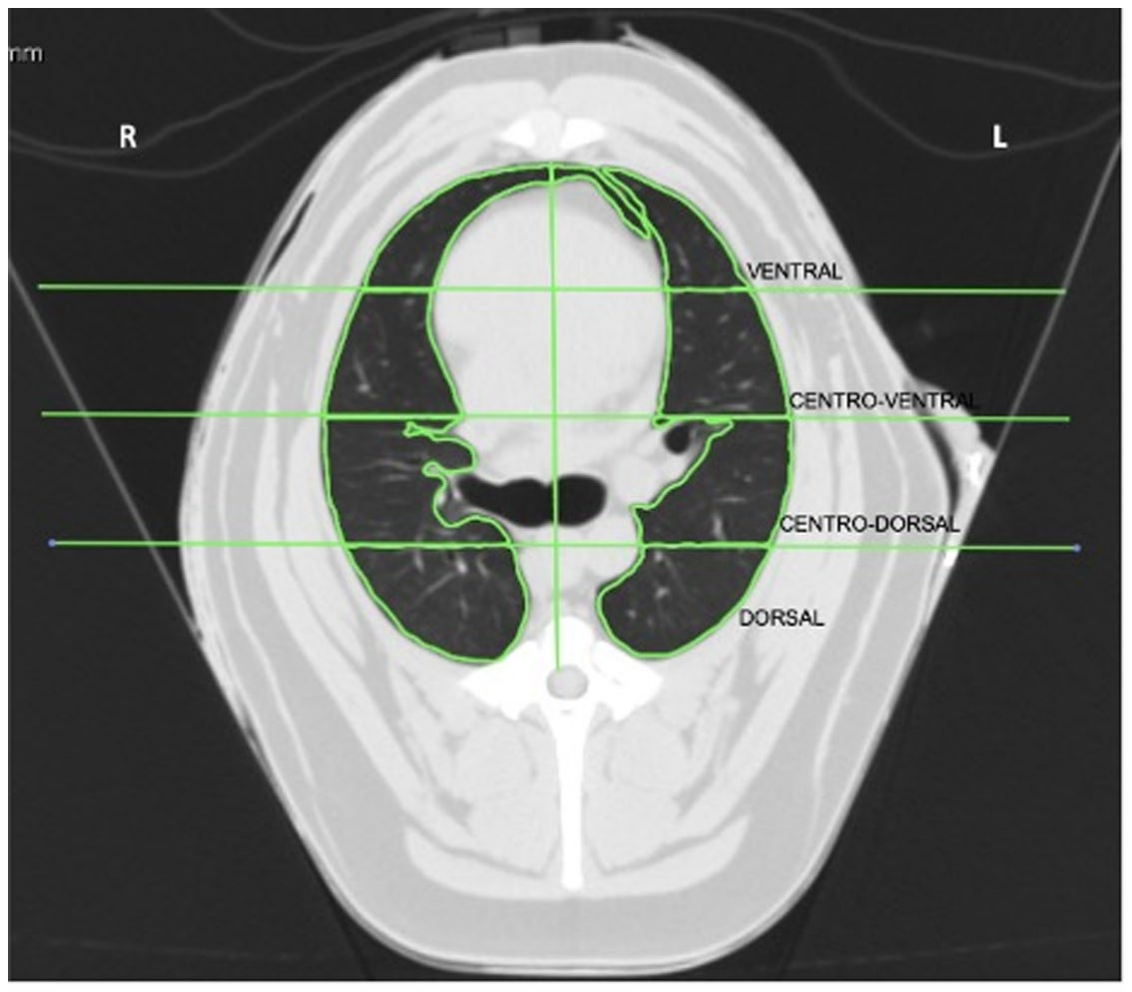
Computed tomography image of lungs at the seventh intercostal space divided into left and right regions of interest (ROI): ventral, central-ventral, central-dorsal, and dorsal. R, right side of the lung; L, left side of the lung.

The acquired images were analyzed using Horos 1.1.7 software (Horos, Purview, Annapolis, EUA). The region selected for the evaluation was at the beginning of the bifurcation of the trachea at the level of the carina. After selection, the cut was divided into four regions of interest (ROI): dorsal, central-dorsal, central-ventral, and ventral. The segmented lungs were manually separated into the left and right lung fields. The accessory lung lobe is included in the right lung field. ROIs were drawn manually, including only the lung tissue, and excluding the bronchi and large vessels (Figure 1).

##### 2.4.1.2 Quantitative CT analysis

Lung ROIs were analyzed to determine the degree of lung attenuation (tissue density) HU. In the present study, a classification scheme was used for the analysis of lung attenuation, as follows: hyperaerated (−1,000 to −901 HU), normally aerated (−901 to −501 HU), poorly aerated (−500 to −101 HU), and non-aerated (−100 to +100) (4).

The regional volumes (RV) and global volumes (GV) were calculated from the values of the pulmonary area and density (HU) of each ROI (13–15) as follows:

RV = Volume ROI X (HU ROI/ -1000), during inspiration and expiration.

where Volume ROI = ROI area x 0.2 cm (cut thickness).

GV = Σ (RV ROI 1,2,3,4 inspiratory + RV ROI 1,2,3,4 expiratory).

#### 2.4.2 Lung mechanics and cardiovascular data

A variable orifice flow sensor (G5, Hamilton Medical, Bonaduz, Switzerland) was calibrated before each patient’s ventilation to assess the tidal volume, pressure, and lung segment volume. The static compliance of the respiratory system (Cst) and driving pressure (DP) were calculated as described previously (16). Heart rate (HR) and invasive mean arterial blood pressure (MABP) were evaluated every 5 min using a multiparametric monitoring system (Nihon Kohden Life Scope Triton - Nihon Kohden Corporation., Japan) throughout the study.

### 2.5 Statistical analysis

Sample size calculation^1^ indicated that seven dogs the minimum necessary to have a 90% chance of observing an increase in the pulmonary density in the left dorsal ROI during inspiration from – 586 HU at PEEP 0 cmH_2_O to – 685 HU at PEEP 5 cmH_2_O descending, at a risk of 5%, considering a standard deviation (SD) of – 70 HU. However, five more dogs were included to increase the chances of obtaining the results. The data used in this calculation were extracted from a pilot study.

Data were assessed for normality using the Shapiro-Wilk test before test selection and calculated and reported as mean and standard deviation (SD). Time influence was analyzed using two-way analysis of variance, followed by a *post-hoc* Bonferroni test to identify differences between time points. Statistical significance was attributed to a value of *p* of <0.05. Statistical analysis were performed using GraphPad Prism 6 for Windows (GraphPad Software, Boston, Massachusetts, USA).

## 3 Results

### 3.1 Animal characteristics

A total of 12 dogs of different breeds were studied, weighing 24.0 ± 6.0 kg, aged 3 ± 1 years, five males and seven females.

### 3.2 Outcome of ARMs in CT data

#### 3.2.1 Lung attenuation

In the right lungs, there was a decrease in lung attenuation in all ROIs compared to PEEP 0 cmH_2_O during inspiration and expiration. In the ventral ROI, a decrease in lung attenuation occurred from PEEP 10 cmH_2_O ascending (*p* = 0.0019) to PEEP 5 cmH_2_O descending (*p* = 0.0021) during inspiration and from PEEP 5 cmH_2_O ascending (*p* = 0.0498) to PEEP 5 cmH_2_O descending (*p* < 0.0001) during expiration. In the central-ventral ROI, there was a decrease in lung attenuation from PEEP 5 cmH_2_O ascending (*p* = 0.0014) to PEEP 5 cmH_2_O descending (*p* < 0.0001) during inspiration and from PEEP 5 cmH_2_O ascending to PEEP 5 cmH_2_O descending (*p* < 0.0001) during expiration. Similarly, the central-dorsal ROI showed a decrease in lung attenuation from PEEP 5 cmH_2_O ascending (*p* = 0.0038) to PEEP 5 cmH_2_O descending (*p* = 0.0110) during inspiration and from PEEP 5 cmH_2_O ascending to PEEP 5 cmH_2_O descending (p < 0.0001) during expiration. Similarly, there was a decrease in lung attenuation in the dorsal ROI from PEEP 5 cmH_2_O ascending (*p* = 0.0064) to PEEP 5 cmH_2_O descending (*p* = 0.0014) during inspiration and from PEEP 5 cmH_2_O ascending to PEEP 5 cmH_2_O descending (*p* < 0.0001) during expiration (Table 1; Supplementary Figure 1).

**Table 1:** Right lung attenuation data presented by means and standard deviation of the ROIs of the right lungs, evaluated by CT of the 12 animals submitted to ARMs during mechanical ventilation.

ROI	PEEP 0	PEEP 5i	PEEP 10i	PEEP 15i	PEEP 20	PEEP15d	PEEP10d	PEEP 5d
Ventral R insp	−802 ± 54	−831 ± 43	−857 ± 23*	−876 ± 25*	−873 ± 28*	−876 ± 23*	−864 ± 29*	−857 ± 22*
Ventral R exp	−769 ± 73	−808 ± 52*	−853 ± 28*	−867 ± 19*	−877 ± 22*	−863 ± 27*	−864 ± 28*	−846 ± 37*
Central-ventral R insp	−783 ± 38	−824 ± 33*	−845 ± 22*	−856 ± 20*	−873 ± 22*	−862 ± 25*	−849 ± 24*	−835 ± 26*
Central-ventral R exp	−743 ± 35	−796 ± 28*	−826 ± 21*	−848 ± 24*	−863 ± 19*	−861 ± 28*	−839 ± 22*	−804 ± 31*
Central-dorsal R insp	−719 ± 44	−762 ± 35*	−794 ± 29*	−825 ± 26*	−858 ± 21*	−840 ± 23*	−815 ± 34*	−772 ± 30*
Central-dorsal R exp	−594 ± 61	−666 ± 55*	−743 ± 44*	−787 ± 40*	−834 ± 29*	−812 ± 35*	−766 ± 38*	−688 ± 52*
Dorsal R insp	−663 ± 46	−716 ± 42*	−772 ± 34*	−808 ± 34*	−845 ± 23*	−827 ± 27*	−789 ± 36*	−723 ± 41*
Dorsal R exp	−590 ± 52	−665 ± 49*	−740 ± 43*	−782 ± 39*	−831 ± 26*	−808 ± 32*	−761 ± 34*	−681 ± 48*

Right lung attenuation data. Data are expressed as a mean and standard deviation; *: statistically significantly different from baseline (PEEP 0 cmH_2_O). ROI, region of interest; PEEP 0, without PEEP; PEEP 5i, PEEP 5 cmH_2_O increasing; PEEP 10i, PEEP 10 cmH_2_O increasing; PEEP 15i, PEEP 15 cmH_2_O increasing; PEEP 20, PEEP 20 cmH_2_O increasing; PEEP 15d, PEEP 15 cmH_2_O decreasing; PEEP 10d, PEEP 10 cmH_2_O decreasing; PEEP 5d, PEEP 5 cmH_2_O decreasing. Ventral R insp and ventral R exp, right ventral ROI during inspiration and expiration. Central-ventral R insp and central-ventral R exp: right central-ventral ROI during inspiration and expiration. Central-dorsal R insp and central-dorsal R exp: right central-dorsal ROI during inspiration and expiration. Dorsal R insp and dorsal R exp: right dorsal ROI during inspiration and expiration.

In the left lungs, similar to the right lung, there was a decrease in lung attenuation in all ROIs compared with PEEP 0 cmH_2_O during inspiration and expiration. During inspiration, a significant reduction in lung attenuation occurred in the ventral ROI, from PEEP 15 cmH_2_O ascending (*p* = 0.0146) to PEEP 10 cmH_2_O descending (*p* = 0.0485). During expiration, in the same ROI, lung attenuation decreased from PEEP 15 cmH_2_O ascending (*p* = 0.0384) to PEEP 10 cmH_2_O descending (*p* = 0.0079). In central-ventral ROI, there was a decrease in lung attenuation from PEEP 15 cmH_2_O ascending (*p* = 0.0459) to PEEP 15 cmH_2_O descending (*p* = 0.0331) during inspiration and from PEEP 10 cmH_2_O ascending (*p* = 0.0013) to PEEP 5 cmH_2_O descending (*p* = 0.008) during expiration. However, in the central-dorsal ROI, a decrease in lung attenuation occurred from PEEP 5 cmH_2_O ascending to PEEP 5 cmH_2_O descending (*p* = 0.0038) during inspiration and from PEEP 5 cmH_2_O ascending (*p* < 0.0001) to PEEP 5 cmH_2_O descending (*p* = 0.0016) during expiration. Similarly, there was a decrease in lung attenuation in the dorsal ROI from PEEP 5 cmH_2_O ascending (p < 0.0001) to PEEP 5 cmH_2_O descending (*p* = 0.0002) during inspiration and from PEEP 5 cmH_2_O ascending to PEEP 5 cmH_2_O descending (p < 0.0001) during expiration (Table 2).

**Table 2:** Left lung attenuation data presented by means and standard deviation of the ROIs of the left lungs, evaluated by CT of the 12 animals submitted to ARMs during mechanical ventilation.

ROI	PEEP 0	PEEP 5i	PEEP 10i	PEEP 15i	PEEP 20	PEEP15d	PEEP10d	PEEP 5d
Ventral L insp	−774 ± 73	−802 ± 78	−814 ± 52	−855 ± 22*	−862 ± 24*	−854 ± 27*	−838 ± 51*	−815 ± 56
Ventral L exp	−745 ± 68	−798 ± 58	−818 ± 56	−850 ± 26*	−855 ± 24*	−841 ± 51*	−845 ± 24*	−800 ± 38
Central-ventral L insp	−661 ± 192	−701 ± 185	−787 ± 48	−823 ± 32*	−854 ± 26*	−847 ± 27*	−818 ± 32	−788 ± 43
Central-ventral L exp	−679 ± 59	−733 ± 59	−768 ± 57*	−815 ± 36*	−850 ± 18*	−832 ± 33*	−811 ± 31*	−773 ± 35*
Central-dorsal L insp	−660 ± 94	−708 ± 77*	−771 ± 52*	−814 ± 32*	−853 ± 16*	−838 ± 28*	−802 ± 36*	−750 ± 45*
Central-dorsal L exp	−601 ± 82	−663 ± 81*	−735 ± 71*	−794 ± 40*	−844 ± 22*	−820 ± 27*	−784 ± 41*	−707 ± 51*
Dorsal L insp	−601 ± 82	−663 ± 81*	−735 ± 71*	−794 ± 40*	−844 ± 22*	−820 ± 27*	−784 ± 41*	−707 ± 51*
Dorsal L exp	−515 ± 83	−600 ± 82*	−674 ± 86*	−763 ± 52*	−827 ± 26*	−801 ± 29*	−750 ± 45*	−665 ± 44*

Data are expressed as a mean and standard deviation; *: statistically significantly different from baseline (PEEP 0 cmH_2_O). ROI, region of interest; PEEP 0, without PEEP; PEEP 5i, PEEP 5 cmH_2_O increasing; PEEP 10i, PEEP 10 cmH_2_O increasing; PEEP 15i, PEEP 15 cmH_2_O increasing; PEEP 20, PEEP 20 cmH_2_O increasing; PEEP 15d, PEEP 15 cmH_2_O decreasing; PEEP 10d, PEEP 10 cmH_2_O decreasing; PEEP 5d, PEEP 5 cmH_2_O decreasing. Ventral L insp and Ventral L exp, left ventral ROI during inspiration and expiration. Central-ventral L insp and central-ventral L exp, left central-ventral ROI during inspiration and expiration. Central-dorsal L insp and central-dorsal L exp, left central-dorsal ROI during inspiration and expiration. Dorsal L insp and dorsal L exp, left dorsal ROI during inspiration and expiration.

#### 3.2.2 Regional volume

##### 3.2.2.1 Right lung

**Figure 2:**
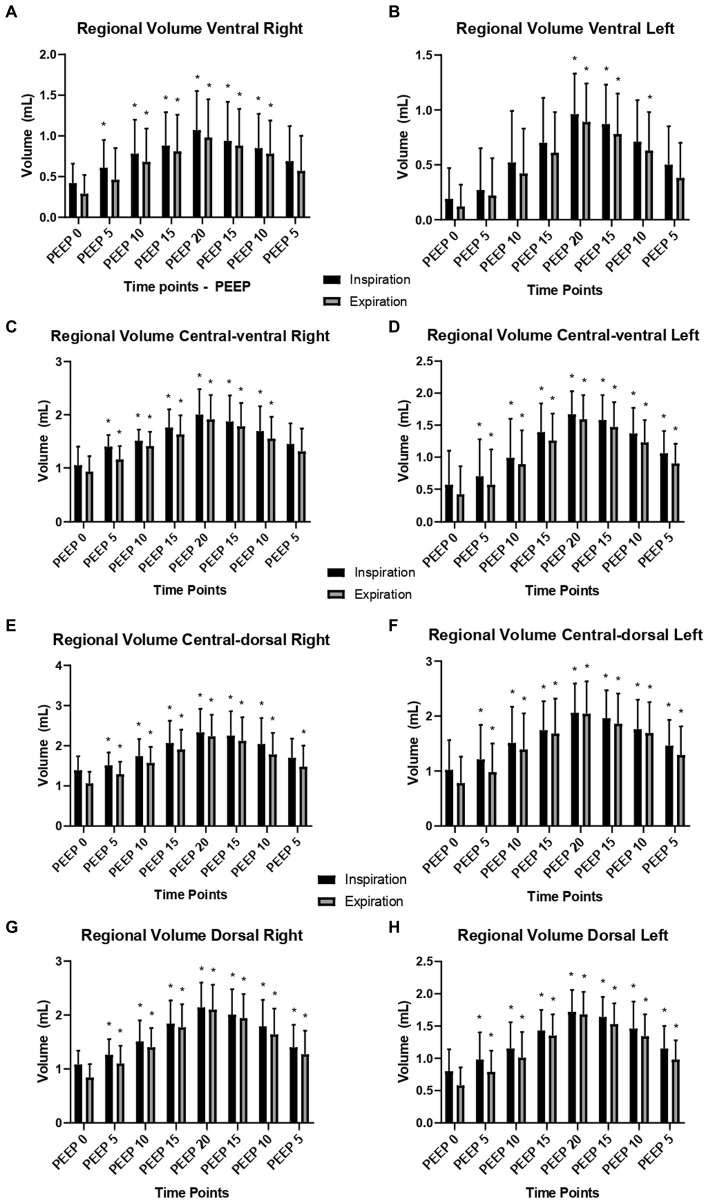
Right and left regional lung volumes according to different ROIs: means and standard deviation of the regional volumes (ml) of the right and left lung evaluated by CT, of the 12 animals submitted to ARM during mechanical ventilation. **(A)** Regional volume of ROI ventral right; **(B)** Regional volume of ROI ventral left; **(C)** Regional volume of ROI central-ventral right; **(D)** Regional volume of ROI central-ventral left; **(E)** Regional volume of ROI central-dorsal right; **(F)** Regional volume of ROI central-dorsal left; **(G)** Regional volume of ROI dorsal right; **(H)** Regional volume of ROI dorsal left. Black bars: inspiratory regional volume. Grey bars: expiratory regional volume. * Differs from PEEP 0 (*p* < 0.05).

Regarding the ventral ROI, there was an increase in the regional gas volume from PEEP 15 cmH_2_O ascending (*p* = 0.0374) to PEEP 15 cmH_2_O descending (*p* = 0.0131) during inspiration and from 15 cmH_2_O ascending (*p* = 0.0141) to PEEP 10 cmH_2_O descending (*p* = 0.0223) during expiration, compared to PEEP 0 cmH_2_O. In the central-ventral ROI, there was an increase in the regional gas volume from PEEP 10 cmH_2_O ascending (*p* = 0.0176) to PEEP 10 cmH_2_O descending (*p* = 0.0003) during inspiration and from PEEP 10 cmH_2_O ascending (*p* = 0.0132) to PEEP 10 cmH_2_O descending during expiration, compared to PEEP 0 cmH_2_O. There were no significant differences between inspiration and expiration (Figure 2; Supplementary Table 1).

Similarly, the central-dorsal ROI showed an increase in the regional gas volume from PEEP 15 cmH_2_O ascending (*p* = 0.0055) to PEEP 10 cmH_2_O descending (*p* = 0.0095) during inspiration and expiration (*p* = 0.0002, *p* = 0.0025), compared to PEEP 0 cmH_2_O. Similarly, the dorsal ROI increased from PEEP 15 cmH_2_O ascending (*p* = 0.0001) to PEEP 10 cmH_2_O descending (*p* = 0.0002) during inspiration and expiration (*p* = 0.0056, *p* = 0.0001), compared to PEEP 0 cmH_2_O. There were no significant differences between inspiration and expiration (Figure 2).

##### 3.2.2.2 Left Lung

Regarding the ventral ROI, there was an increase in the regional gas volume from PEEP 15 cmH_2_O ascending (*p* = 0.0048) to PEEP 10 cmH_2_O descending (*p* = 0.0037) during inspiration and from PEEP 15 cmH_2_O ascending (*p* = 0.0173) to PEEP 10 cmH_2_O descending during expiration, compared to PEEP 0 cmH_2_O. Similarly, in the central-ventral ROI, there was an increase in the regional gas volume from PEEP 15 cmH_2_O ascending to PEEP 10 cmH_2_O descending (*p* = 0.0001) during inspiration and from PEEP 15 cmH_2_O ascending (*p* = 0.0001) to PEEP 10 cmH_2_O descending (*p* = 0.0001) during expiration, compared to PEEP 0 cmH_2_O. There were no significant differences between inspiration and expiration (Figure 2; Supplementary Table 2).

The central-dorsal and dorsal ROIs presented an increase in regional gas volume. In the central-dorsal ROI, the increase occurred from PEEP 15 cmH_2_O ascending (*p* = 0.0118) to PEEP 10 cmH_2_O descending (*p* = 0.0095) during inspiration, and from PEEP 15 cmH_2_O ascending (*p* = 0.0009) to PEEP 10 cmH_2_O descending (*p* = 0.0008) during expiration, compared to PEEP 0 cmH_2_O. In the dorsal ROI, the increase occurred from PEEP 15 cmH_2_O ascending (*p* = 0.0001) to PEEP 10 cmH_2_O descending (p = 0.0095) during inspiration and from PEEP 15 cmH_2_O ascending (*p* = 0.0185) to PEEP 10 cmH_2_O descending (*p* < 0.0001) during expiration, compared to PEEP 0 cmH_2_O. There were no significant differences between inspiration and expiration (Figure 2).

#### 3.2.3 Global volume

There was a significant increase in the global volume of gas during inspiration and expiration in the right and left lung sides compared with PEEP 0 cmH_2_O. The global volume in the right lung increased from PEEP 5 cmH_2_O ascending (*p* = 0.0001) to PEEP 5 cmH_2_O descending (*p* = 0.01) during inspiration and expiration and from PEEP 5 cmH_2_O ascending (*p* = 0.0001) to PEEP 5 cmH_2_O descending (*p* < 0.01), compared with PEEP 0 cmH_2_O. The same pattern was observed in the left lung. No significant differences were observed between inspiration and expiration (Table 3).

**Table 3:** Means and standard deviation of the global volumes (ml) of the left and right lungs, evaluated by CT, of the 12 animals submitted to ARMs during mechanical ventilation.

ROI	PEEP 0	PEEP 5i	PEEP 10i	PEEP 15i	PEEP 20	PEEP 15d	PEEP 10d	PEEP 5d
GV R Insp	4.03 ± 0.77	4.77 ± 0.55 *	5.55 ± 1.12*	6.55 ± 1.46*	7.53 ± 1.77*	7.07 ± 1.86*	6.37 ± 1.85*	5.25 ± 1.52*
GV R Exp	3.11 ± 0.82	4.01 ± 1.01*	5.05 ± 1.14*	6.11 ± 1.53*	7.22 ± 1.68*	6.72 ± 1.74*	5.76 ± 1.70*	4.64 ± 1.61*
GV L Insp	2.57 ± 1.58	3.16 ± 1.81*	4.17 ± 1.90*	5.25 ± 1.48*	6.41 ± 1.32*	6.05 ± 1.37*	5.30 ± 1.50*	4.17 ± 1.29*
GV L Exp	1.90 ± 1.32	2.56 ± 1.62*	3.70 ± 1.74*	4.90 ± 1.53*	6.19 ± 1.38*	5.64 ± 1.34*	4.89 ± 1.42*	3.54 ± 1.26*

GV R Insp, global volume of the right lung at the inspiratory moment; GV R Exp, global volume of the right lung at the expiratory moment; GV L Insp, global volume of the left lung at the inspiratory moment; GV L Exp, global volume of the left lung at the expiratory moment; * differs from PEEP 0 (*p* < 0.05).

#### 3.2.4 Lung mechanics and cardiovascular data

**Figure 3:**
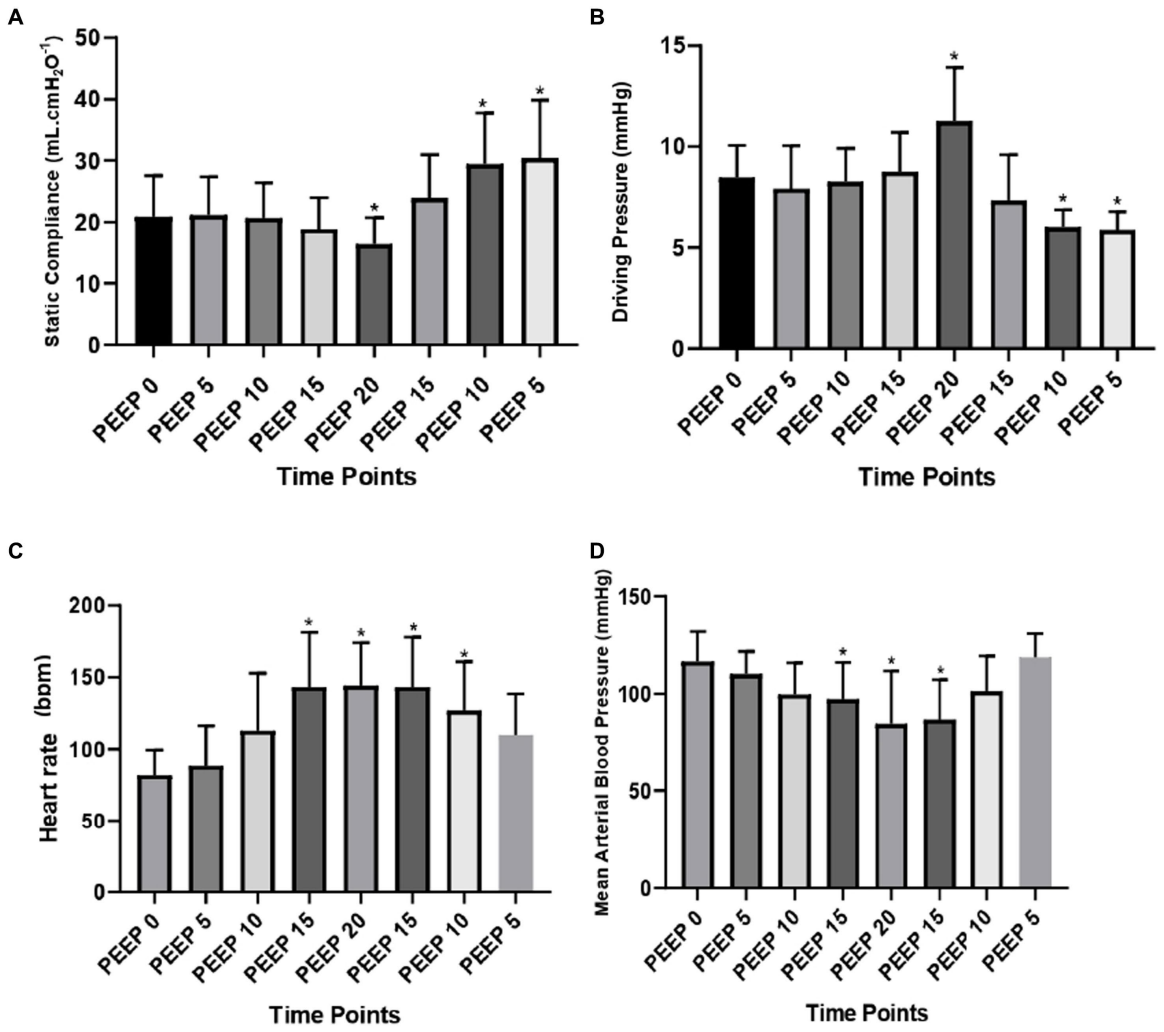
Lung mechanics and cardiovascular data: means and standard deviation of the static compliance of the respiratory system (ml. cmH_2_O^−1^) **(A)**; driving pressure (cmH_2_O) **(B)**; heart rate (bpm) **(C)**; and mean arterial blood pressure (mmHg) **(D)** evaluated by CT of the 12 animals submitted to ARMs during mechanical ventilation. * Differs from PEEP 0 (*p* < 0.05).

Regarding the Cst, there was a decrease at PEEP 20 cmH_2_O compared to PEEP 0 (from 20.9 ± 6.6 to 16.5 ± 4.3 mL. cmH_2_O^−1^; *p* = 0.0015) and an increase at PEEP 10 cmH_2_O descending (from 20.9 ± 6.6 to 29.5 ± 8.2 mL. cmH_2_O^−1^; *p* = 0.0003), and PEEP 5 cmH_2_O descending (from 20.9 ± 6.6 to 30.4 ± 9.5 mL. cmH_2_O^−1^; *p* = 0.0001). On the other hand, the DP increased at PEEP 20 cmH_2_O (from 8.6 ± 1.5 to 12.1 ± 1.7 cmH_2_O; *p* = 0.039) and decreased at PEEP 10 cmH_2_O descending (from 8.6 ± 1.5 to 6.2 ± 0.6 cmH_2_O; *p* = 0.0007), and PEEP 5 cmH_2_O descending (from 8.6 ± 1.5 to 6.2 ± 0.4 cmH_2_O; *p* = 0.0008) compared to PEEP 0 cmH_2_O (Figure 3).

The HR (bpm) increased from PEEP 15 cmH_2_O ascending (from 82.1 ± 17.4 to 143.3 ± 38.3; *p* = 0.0021) to PEEP 10 cmH_2_O descending (from 82.1 ± 17.4 to 127.1 ± 33.8; *p* = 0.0061) compared to PEEP 0 cmH_2_O. However, the MABP decreased at PEEP 15 cmH_2_O ascending (from 69.4 ± 9.5 to 65.8 ± 10.7; *p* = 0.0106), with a maximum decrease at PEEP 20 cmH_2_O (from 69.4 ± 9.5 to 60.8 ± 19.7; *p* = 0.0001) and at PEEP 15 cmH_2_O descending (from 69.4 ± 9.5 to 63.0 ± 12.9; *p* = 0.0018) compared to PEEP 0 cmH_2_O (Figure 3).

## 4 Discussion

The study hypothesis was not supported. During the PEEP 20 cmH_2_O, the lungs were normoaerated, and the nondependent regions were not overdistended. As expected, a peep 5 cmH_2_O descending did not promote atelectasis.

The main finding was reduced pulmonary attenuation throughout the ARM compared to PEEP 0 cmH_2_O and increased regional and global volume on both sides of the lungs. The maximum increase in volumes occurred at PEEP 20 cmH_2_O, followed by PEEP 15, 10, and 5 cmH_2_O descending. Alveolar recruitment was maintained until the end of the maneuver because PEEP 5 cmH_2_O descending was higher than PEEP 0 cmH_2_O. Another important finding was that even at the highest PEEP value, the lungs remained normoaerated, with the highest density of - 877 HU at the ventral right ROI during expiration and -862 HU at the ventral left ROI during inspiration. Regarding respiratory mechanics, the Cst increased significantly at PEEP 10 and 5 cmH_2_O descending, and DP decreased simultaneously. The hemodynamics parameters showed increased HR and a decreased in MABP at the highest PEEP values (PEEP 15i, 20,15d cmH_2_O).

As is already known, anesthesia and recumbency promote the closure of alveolar units, as demonstrated by tomography as a significant increase in attenuation and reduction in lung volume, especially in dependent areas. Similarly, in this study, the dependent regions (central-dorsal and dorsal) showed a greater increase in attenuation at PEEP 0 cmH_2_O, which was compatible with a reduction in aeration in these areas. However, the values obtained cannot be considered atelectasis because they were between −900 and −501 HU. A reduction in aeration was expected, considering that the animals were ventilated for the initial 30 min with a tidal volume of 7 mL.kg^−1^, PEEP 0 cmH_2_O, FiO_2_ 1.0. According to Staffieri et al. (9) and De Monte et al. (11), dogs ventilated with FiO_2_ of 1.0 in dorsal decubitus for 40 min, with a tidal volume of 12 to 15 mL.kg^−1^ showed larger areas of atelectasis in the lung-dependent regions, as confirmed by CT images. They observed that when FiO_2_ was 1.0 compared to 0.40, there was a 10% increase in the non-aerated region, 9.2% in the poorly aerated region, and a reduction of 18.2% in normoaerated lung regions. Oxygen is a highly diffusible gas that promotes partial or complete alveolar collapse in areas of the lungs (11, 17). A tidal volume of 7 mL.kg^−1^ is considered low tidal volume in humans and dogs and, when associated with zero end-expiratory pressure, it can promote alveolar collapse (18). However, in the present study, CT did not reveal atelectasis, probably because the time spent under this condition was insufficient to promote alveolar collapse.

The observed increase in gas volume during the ARM was similar to the findings of Bugedo et al. (19). They evaluated two recruitment maneuvers in patients with acute respiratory distress syndrome (ARDS) using CT. They verified that, during the recruitment maneuver, the tidal volume increased mainly at higher PEEP values (PEEP 30 cmH_2_O), and even when PEEP returned to a lower level, the tidal volume was still higher than the baseline. The same was observed in the present study in all regions and was reflected by an increase in lung aeration in the lungs of healthy dogs. An increase in the regional volume was evident in all ROIs, mainly in the dependent areas (central-dorsal and dorsal) of both lungs, during inspiration and expiration, demonstrating that the lveolar units were recruited.

According to the attenuation differences classification, it was found that the lungs remained normally aerated throughout the recruitment, and the ARM did not promote hyperdistention. Although ARM by PEEP titration improves regional compliance of the dependent portions of the lung associated with a more homogeneous distribution of aeration, higher levels of PEEP can be associated with lower compliance of nondependent lung regions, which could indicate overdistention (20). However, the CT scans played an important role, helping to monitor the ARM, evaluating hypertension, and stretching the alveolar units (quantified by the increase in attenuation from −900 to –1000 HU). In contrast, Ambrósio et al. (20), assessing dogs under the same tidal volume and ARM, using electrical impedance tomography to assess the maneuver, and observed hyperdistention at PEEP 20 cmH_2_O in the nondependent ROI and increased regional compliance of the dependent ROI. These findings are probably due to the different techniques employed to evaluate ARMs.

The inversely proportional relationship between the plateau pressure difference with PEEP and the tidal volume provides the static compliance of the respiratory system at different times (21). At the application of higher PEEP (20 cmH_2_O), the lowest Cst value was observed because of the ratio between the same tidal volume and the higher pressure applied. However, at the end of the ARM, with a decrease in PEEP to 5 cmH_2_O, the best Cst value was observed because the same tidal volume resulted in lower airway pressure, indicating alveolar opening compared to the beginning of the experimental protocol. According to De Monte et al. (11), in dogs, PEEP 5 cmH_2_O maintained the alveoli open after ARM. Similarly, Ambrósio et al. (21) observed that the best PEEP in the dogs after ARM by PEEP titration was between 10 and 5 cmH_2_O descending. Di Bella et al. (22) verified that PEEP 5 cmH_2_O could also maintain the ARM in healthy dogs undergoing laparoscopy. In addition, Rodrigues et al. (23) concluded that PEEP 5 cmH_2_O was sufficient to support the alveoli open with or without ARM in dogs undergoing 1 h of dental surgery and using low tidal volume (8 mL.kg^−1^).

Driving pressure is considered one of the most critical factors in lung protection, and is associated with tidal volume and moderate PEEP during mechanical ventilation in human beings. This variable is strongly associated with ICU patient survival, as its reduction is related to lung injuries and, consequently, more ventilated access. According to Amato et al. (16), PEEP and low tidal volume increments are beneficial when associated with reduced DP. In humans with ARDS, the DP value must be below 15 cmH_2_O, but determining the best values in dogs is still necessary. Bugedo et al. (24) suggest using a tidal volume between 6 and 8 mL.kg^−1^, moderate PEEP levels, and ventilation adjustment according to driving pressure, which should be lower than 15 cmH_2_O in injured human lungs. In patients without lung injury, DP is likely to be less than 10 cmH_2_O with a normal or close-to-normal Cst. In this study, the initial DP at PEEP 0 cmH_2_O was 8 cmH_2_O, reaching 12 cmH_2_O only at the highest PEEP (20 cmH_2_O) when Cst also worsened. When PEEP values returned to 10 and 5 cmH_2_O descending, the DP decreased to 6 cmH_2_O, and the Cst also showed improvement. Therefore, when the DP decreases and Cst increases, this demonstrates that at low pressure, the volume expands the lungs adequately, and that can be the best value of PEEP to keep the alveoli open.

The negative impact of ARM on hemodynamic variables is anticipated due to intrathoracic pressure increases; however, the effects are transitory, and hemodynamics return to baseline values after a few minutes depending on the patient’s volemic status (22, 25). According to Nielsen et al. (26), the hemodynamic effects of ARMs are more pronounced in hypovolemic patients than in normovolemic patients. However, in the current study, there was a significant increase (74%) in HR when PEEP reached 15 cmH_2_O ascending and a reduction in MABP (13.5%) when PEEP reached 20 cmH_2_O, and values returned close to values of PEEP 0 cmH_2_O in 10 min. The same changes were observed by Canfran et al. (27) in beagles undergoing ARMs, where PEEP titration, which were minimized when a bolus of fluid therapy was administered before the maneuvers. Similarly, Ambrósio et al. (20) observed an increase in HR in dogs undergoing the same ARM at the same values of PEEP, which returned to baseline values 5 min after the end of the maneuver, and MABP did not change. The authors observed similar hemodynamic results in dogs and humans undergoing cardiac and echocardiographic studies (28–32). Therefore, it is essential to emphasize that care must be taken with high values of intrathoracic pressure during ARMs.

### 4.1 Limitations

The main limitation of this study was that the assessment of alveolar recruitment was performed in healthy lungs, which reduced the observation of its effects. Another limitation is that the best value of PEEP can change depending on the time it is evaluated after ARMs. The alveoli become de-recruited over time because of compression or absorption atelectasis. This study only assessed the lung area in the seventh intercostal space. Images were selected beginning with the bifurcation of the trachea at the level of the carina, which may not reflect the most posterior regions of the lungs.

## 5 Conclusion

Lung attenuation and regional and global volumes assessed by CT showed that the maximum pulmonary aeration distribution followed by PEEP titration occurred at PEEP 20 cmH_2_O, maintaining the lungs with normoaeration and without producing hyperaeration. Alveolar recruitment was maintained even in descending PEEP 10 and 5 cmH_2_O, as demonstrated by the maintenance of volumes higher than the PEEP 0 cmH_2_O. Based on the best Cst and DP associated with the absence of hemodynamic changes, the best PEEP value to keep the alveoli open after ARMs was PEEP from 10 to 5 cmH_2_O descending in this study condition and for volume-controlled ventilation.

## Data availability statement

The original contributions presented in the study are included in the article/Supplementary material, further inquiries can be directed to the corresponding author.

## Ethics statement

The animal studies were approved by CEUA- FMVZ-USP: Ethics Committee on the Use of Animals at the Faculty of Veterinary Medicine and Animal Science at the University of São Paulo – Brazil. The studies were conducted in accordance with the local legislation and institutional requirements. Written informed consent was obtained from the owners for the participation of their animals in this study.

## Author contributions

AA designed the study, performed the experimental procedures, interpreted the data, performed the statistics, and wrote the manuscript. AS, MP, FA, and RR participated in the study design and experimental procedure. AF, CB, and BF participated in data CT acquisition. DF performed the experimental methods and critically reviewed the manuscript. All authors contributed to the critical revision of the manuscript and approved the final manuscript.

## Footnotes

^1^https://www.clinicalc.com/stats/samplesize.aspx
