# Supplementary material for: Effects of a stepwise alveolar recruitment maneuver on lung volume distribution in dogs assessed by computed tomography
Source: Front Vet Sci. 2024 Jan 16;10:1232635. doi: 10.3389/fvets.2023.1232635 (PMC10825017; doi:10.3389/fvets.2023.1232635)
Supplement: Supplementary file 1 [file Data_Sheet_1.pdf]

Supplementary Material

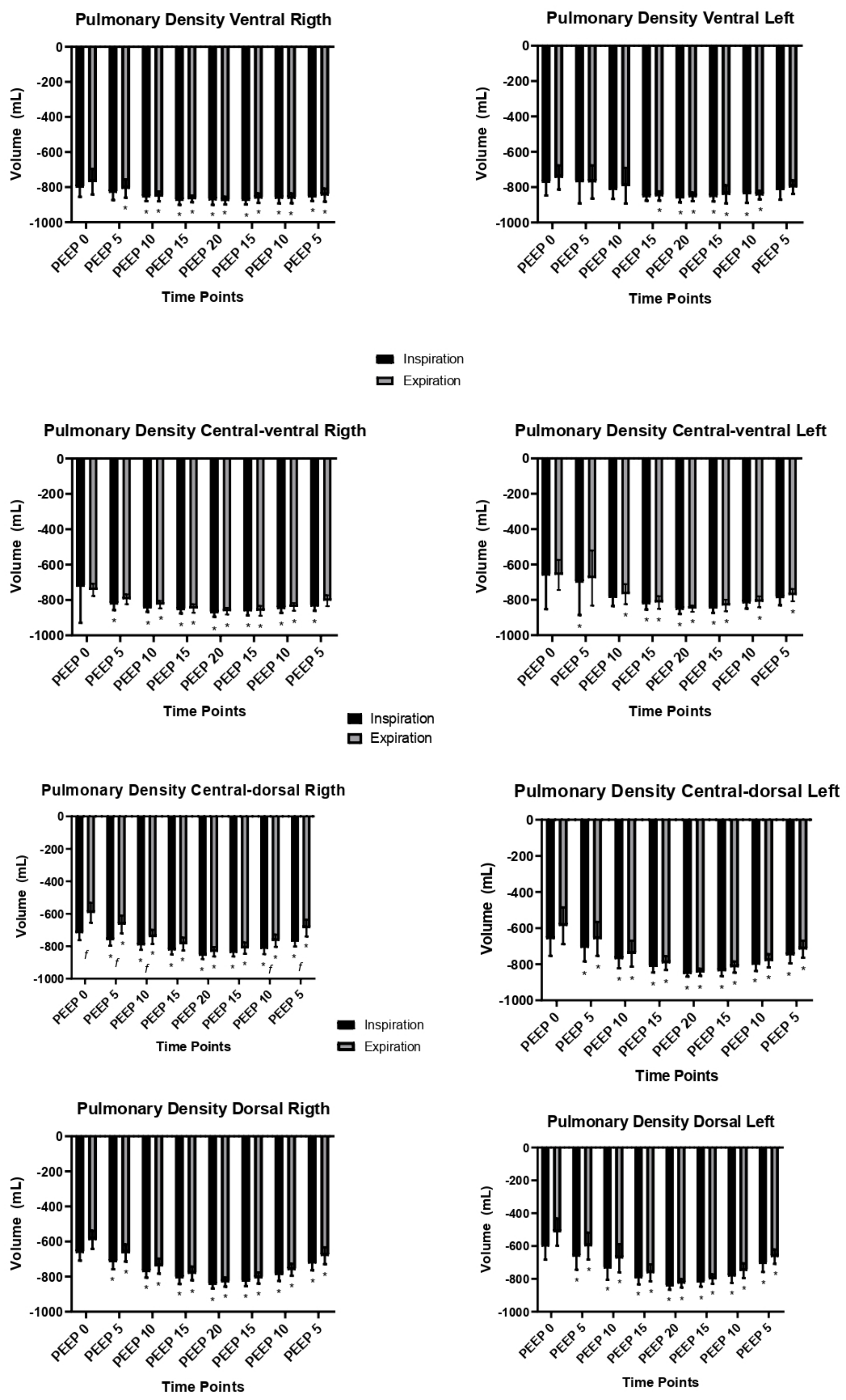

Supplementary Figure 1: Lung Attenuation Data: means and standard deviation of the lung density (HU) of the right and left lung evaluated by CT, of the 12 animals submitted to ARM during mechanical ventilation. Black bars: inspiratory density. Grey bars: expiratory density. * Differs from PEEP 0 ($p<0.05$).

Supplementary Table 1: Means and standard deviation of the right lung regional volumes (ml), evaluated by CT, of the 12 animals submitted to ARM during mechanical ventilation

ROI	PEEP 0	PEEP 5i	PEEP 10i	PEEP 15i	PEEP 20	PEEP 15d	PEEP 10d	PEEP 5d
**Ventral R insp**	0.42±0.24	0.61±0.34	0.78±0.42	0.88±0.41*	1.07±0.48*	0.94±0.48*	0.85±0.42	0.69±0.43
**Ventral R exp**	0.29±0.23	0.46±0.39	0.68±0.41	0.81±0.45*	0.98±0.47*	0.88±0.45*	0.78±0.41*	0.57±0.43
**Central-ventral R Insp**	1.14±0.19	1.40±0.22	1.51±0.21*	1.76±0.34*	2.00±0.48*	1.87±0.49*	1.69±0.47*	1.45±0.39
**Central-ventral R Exp**	0.93±0.29	1.16±0.25	1.41±0.27*	1.63±0.36*	1.91±0.46*	1.78±0.44*	1.55±0.41*	1.31±0.43
**Central-dorsal R Insp**	1.39±0.35	1.51±0.32	1.74± 0.43	2.07±0.55*	2.33±0.59*	2.25±0.61*	2.04±0.65*	1.70±0.48
**Central-dorsal R Exp**	1.06±0.29	1.29±0.31	1.57± 0.40	1.91±0.49*	2.23±0.54*	2.12±0.59*	1.78±0.54*	1.48±0.52
**Dorsal R Insp**	1.08±0.26	1.26±0.29	1.51±0.39	1.84±0.43*	2.14±0.46*	2.01±0.47*	1.79±0.49*	1.40±0.42
**Dorsal R Exp**	0.84±0.25	1.10±0.33	1.40±0.36*	1.77±0.43*	2.10±0.46*	1.95±0.45*	1.64±0.48*	1.27±0.44

Data are expressed as mean and standard deviation; *: statistically significantly different from PEEP 0 cmH_2O. ROI: region of interest, PEEP 0: without PEEP; PEEP 5i: PEEP of 5 cmH_2O increasing; PEEP 10i: PEEP 10 cmH_2O increasing; PEEP 15i: PEEP 15 cmH_2O increasing; PEEP 20: PEEP 20 cmH_2O increasing; PEEP 15d: PEEP 15 cmH_2O decreasing; PEEP 10d: PEEP 10 cmH_2O decreasing; PEEP 5d: PEEP 5 cmH_2O decreasing. Ventral R insp and Ventral R exp: right ventral ROI during inspiration and expiration. Central-ventral R insp and Central-ventral R exp: right central-ventral ROI during inspiration and expiration. Central-dorsal R insp and Central-dorsal R exp: right central-dorsal ROI during inspiration and expiration. Dorsal R insp and Dorsal R exp: right dorsal ROI during inspiration and expiration.

Supplementary Table 2: Means and standard deviation of the left lung regional volumes (ml), evaluated by CT, of the 12 animals submitted to ARM during mechanical ventilation

ROI	PEEP 0	PEEP 5i	PEEP 10i	PEEP 15i	PEEP 20	PEEP 15d	PEEP 10d	PEEP 5d
**Ventral L Insp**	0.19±0.28	0.26±0.38	0.52±0.47	0.70±0.41*	0.96±0.37*	0.87±0.36*	0.71±0.38*	0.50±0.35
**Ventral L Exp**	0.12±0.20	0.22±0.34	0.41±0.42	0.61±0.37*	0.89±0.35*	0.78±0.37*	0.63±0.35*	0.38±0.32
**Central-ventral L Insp**	0.57±0.53	0.70±0.58	0.99±0.61	1.39±0.45*	1.67±0.36*	1.58±0.39*	1.37±0.40*	1.06±0.35
**Central-ventral L Exp**	0.42±0.44	0.56±0.56	0.89±0.53	1.26±0.42*	1.59±0.38*	1.47±0.39*	1.23±0.35*	0.90±0.31
**Central-dorsal L Insp**	1.02±0.54	1.21±0.63	1.51±0.66	1.74±0.53*	2.06±0.53*	1.96±0.51*	1.76±0.54*	1.46±0.47
**Central-dorsal L Exp**	0.78±0.48	0.98±0.52	1.39±0.66	1.68±0.64*	2.04±0.59*	1.86±0.55*	1.69±0.56*	1.29±0.52
**Dorsal L Insp**	0.80±0.34	0.98±0.42	1.15±0.41	1.43±0.32*	1.72±0.34*	1.64±0.31*	1.46±0.42*	1.15±0.35
**Dorsal L Exp**	0.58±.28	0.79±0.33	1.01±0.40	1.35±0.33*	1.68±0.35*	1.53±0.32*	1.34±0.34*	0.98±0.30

Data are expressed as mean and standard deviation; *: statistically significantly different from PEEP 0 cmH_2O. ROI: region of interest, PEEP 0: without PEEP; PEEP 5i: PEEP of 5 cmH_2O increasing; PEEP 10i: PEEP 10 cmH_2O increasing; PEEP 15i: PEEP 15 cmH_2O increasing; PEEP 20: PEEP 20 cmH_2O increasing; PEEP 15d: PEEP 15 cmH_2O decreasing; PEEP 10d: PEEP 10 cmH_2O decreasing; PEEP 5d: PEEP 5 cmH_2O decreasing. Ventral L insp and Ventral L exp: left ventral ROI during inspiration and expiration. Central-ventral L insp and Central-ventral L exp: Left central-ventral ROI during inspiration and expiration. Central-dorsal L insp and Central-dorsal L exp: Left central-dorsal ROI during inspiration and expiration. Dorsal L insp and Dorsal L exp: Left dorsal ROI during inspiration and expiration.
